# The Impact of Intestinal Ultrasound on the Management of Inflammatory Bowel Disease: From Established Facts Toward New Horizons

**DOI:** 10.3389/fmed.2022.898092

**Published:** 2022-05-23

**Authors:** Olga Maria Nardone, Giulio Calabrese, Anna Testa, Anna Caiazzo, Giuseppe Fierro, Antonio Rispo, Fabiana Castiglione

**Affiliations:** ^1^Department of Public Health, University of Naples Federico II, Naples, Italy; ^2^Department of Clinical Medicine and Surgery, University of Naples Federico II, Naples, Italy

**Keywords:** transmural healing, transmural remission, Crohn’s disease, ulcerative colitis, noninvasive monitoring, intestinal ultrasound

## Abstract

Intestinal ultrasound (IUS) plays a crucial role as a non-invasive and accurate tool to diagnose and assess inflammatory bowel disease (IBD). The rationale for using IUS in Crohn’s disease (CD), a transmural disease, is widely acknowledged. While the use of IUS in ulcerative colitis (UC), a mucosal disease, is often underestimated, but, recently, it is increasingly expanding. In the context of a treat-to-target approach, the role of IUS is shifting toward a monitoring tool for predicting response to therapy. Hence, adjusting therapeutic strategies based on IUS response could reduce the burden related to endoscopy and speed the decision process with the ultimate goal to alter the natural course of IBD. Assessment of bowel wall thickness (BWT) is the most reliable IUS measure. However, the development of validated and reproducible sonographic scores to measure disease activity and the identification of parameters of therapeutic response remain relevant issues to implement the daily adoption of IUS in clinical practice. Accordingly, this review focuses on the current literature investigating the impact of IUS on CD with emphasis on the concept of transmural healing (TH) and the main related advantages. We further explore new insights on the role of IUS in UC and its clinical implications.

## Introduction

Crohn’s disease (CD) and ulcerative colitis (UC) belong to inflammatory bowel diseases (IBD). These are chronic, progressive, and disabling diseases whose incidence has dramatically increased during the last 20 years, impacting on the quality of life, social functioning and psychological health (1–3).

Crohn’s disease potentially affects any part of the gastrointestinal tract with three different phenotypes (non-stricturing/non-penetrating, stricturing, and penetrating) (4), and it is characterized by a progressive and transmural involvement of the mucosa that may require intestinal resection for patients with complications or those with intractable disease (5).

Ulcerative colitis is usually limited to the mucosal layer and involves the colon starting from the rectum in a continuous manner (6). It has been shown that extensive, severe disease at diagnosis and early need for corticosteroids are the main factors significantly associated with a higher risk of colectomy ranging from 1 to 5% at 1 year and from 3 to 8% at 5 years after diagnosis (7).

In recent decades, the clinical management of IBD has significantly evolved, moving from a clinical-based strategy toward a concept of “*deep remission”* with the final intent to reduce structural damage, prevent disease progression and improve long-term outcomes for patients.

The Selecting Therapeutic Targets in Inflammatory Bowel Disease (STRIDE) represented a turning point in the medical strategy of IBD. In 2015, the International Organization for the Study of Inflammatory Bowel Diseases (IOIBD) first introduced the concept of *treat-to-target strategy and tight monitoring* (8). Since then, the expanding use of immune modulators and the advent of monoclonal antibodies driven against tumour necrosis factor-alpha (TNF-alpha), interleukin-12/23 (IL-12/23), and integrins on the leukocytes surface have made possible to go beyond these conventional treatment goals of the STRIDE toward more ambitious targets. In addition, new advanced non-invasive tools are becoming widely available, providing new opportunities for tight monitoring.

In this scenario, the STRIDE II updated the STRIDE by introducing new additional targets, such as serum and fecal biomarkers, normalization of quality of life, and prevention of disability. Furthermore, transmural healing (TH) in CD and histologic remission in UC have been recognized as adjunct measures with the assumption that deep remission implicates better outcomes (9).

The utility of intestinal ultrasound (IUS) in the assessment of CD is well established (10, 11), while the literature surrounding its use in UC remains questioned. In the context of a “treat-to-target” strategy and close monitoring, IUS plays a crucial role due to its non-invasiveness. In addition, it has been further recognized as a reliable tool for detecting therapeutic responses as it offers notable advantages with respect to endoscopy in terms of cost, safety profile, and lack of bowel preparation (12, 13).

This narrative review aimed to discuss the recent advances of IUS in the management of IBD, starting from the current evidence in CD and focusing on the concept of TH and its clinical implication, to the expanding role in UC.

## Methods

### Search Strategy and Selection Criteria

A literature search using the PubMed database from January 2000 to March 2022 was made by two independent reviewers (OMN and GC). The search used the following terms: “transmural healing” or “transmural remission” or “radiological remission” and “Crohn’s disease” or “ulcerative colitis” or “inflammatory bowel disease” and “fecal calprotectin” or “non-invasive markers” and “bowel sonography” or “intestinal ultrasound” and “post-operative recurrence.”

We screened the title and abstract of 12,798 articles, followed by a full-text analysis of relevant articles. Of these, a total of 41 articles were considered suitable. We selected randomized controlled studies, prospective or retrospective cohort studies, and systematic reviews with meta-analysis and excluded duplications, abstracts and studies in languages other than English. For each study, we collected authors, journal, country, number of enrolled patients, the definition of TH, clinical outcomes, time of follow-up, TH response rate, TH assessment method, IUS accuracy for post-operative recurrence diagnosis, IUS accuracy, and correlation with non-invasive markers.

## Results

### Cross-Sectional Imaging for Detecting Transmural Healing

Intestinal ultrasound has become an integral part of IBD clinical management in recent years since it accurately determines disease extent, severity, and response to medical therapy (11).

Bowel wall thickness (BWT) of the mucosal and sub-mucosal layers is the most relevant IUS parameter to detect intestinal inflammation. However, additional IUS parameters of transmural activity include increased echogenicity of the sub-mucosal layer, an amplified color Doppler signal indicative of increased vascularity, and proliferation of mesenteric fat (11). Several meta-analyses have investigated the diagnostic accuracy of cross-sectional imaging methods, i.e., magnetic resonance enterography (MRE), CT enterography (CTE), and IUS and they found high accuracy as well as a high agreement between all these tools without any significant differences (14–17). Nevertheless, it is noteworthy that IUS and MRE are radiation-free methods and, thus, a clear advantage is that patients might have less radiation-related risk. However, choosing one examination rather than another one could depend on the operators’ availability, expertise, costs and times required to do these procedures.

In a pioneering study in 2013, our group (18) evaluated the diagnostic accuracy of IUS and MRE to diagnose small bowel CD. We showed that MRE outperformed IUS in defining CD extension (*r* = 0.71). The agreement between both the techniques was high in terms of disease location (*k* = 0.81), while MRE had a fair concordance with IUS for strictures (*k* = 0.71) and abscesses (*k* = 0.88), and it showed better detection of entero-enteric fistulas (*k* = 0.67).

Subsequently, in 2017, the same group confirmed a high agreement between IUS and MRE for assessing TH (*k* = 0.90; *p* < 0.001) (19).

Furthermore, Allocca et al. (17), in a single centre study, including 60 patients, analyzed the accuracy of IUS vs. MRE combined with colonoscopy for assessing localization, increased vascular signal at power Doppler, disease activity (ulcers at colonoscopy) and complications (strictures, fistulas and abscesses). Notably, IUS could be a useful tool for detecting ulcers in patients with CD, since it showed a diagnostic accuracy of 91% for localization of disease and 96% for ulcerations. Concerning the complications, they reported diagnostic accuracy of 81% for strictures, 98% for fistulas, and 96% for abscesses.

Similarly, a large randomized controlled trial conducted in eight United Kingdom hospitals (15) compared the accuracy between MRE and IUS for assessing CDs presence, extension and activity. Notably, they reported significantly higher sensitivity and specificity of MRE for small bowel extent and higher sensitivity for disease presence in respect of IUS. Furthermore, when they evaluated the diagnostic accuracy for colonic disease, they found no significant difference (consistently lower than for small bowel disease) between MRE and IUS, although the latter had greater sensitivity than MRE in newly diagnosed patients (15).

In an attempt to facilitate reliable IUS identification of CD activity, a recent expert consensus through a Delphi method identified four IUS key parameters of inflammation: BWT, bowel wall stratification, hyperemia of the wall assessed by using color Doppler imaging, and inflammatory mesenteric fat. These four variables were included in the final International Bowel Ultrasound Segmental Activity Score (IBUS-SAS) with almost perfect reliability [intra-class correlation coefficient (ICC) 0.97 0.95–0.99, *p* < 0.001]. Significantly, all of them could predict overall disease activity, but BWT and i-fat are required to predict overall disease severity (20).

### Transmural Healing—Toward a Standardized Definition

Based on IUS, TH was defined as a BWT less than or equal to 3 mm (18, 19, 21–29). However, some studies used other sonographic features for full and comprehensive characterization. The several definitions of TH are shown in Table 1.

**TABLE 1:** Definitions of transmural healing (TH).

References and number of patients	Definition of transmural healing based on IUS
Castiglione et al. (18), (*n* = 133)	BWT ≤ 3 mm
Ripollés et al. (22), (*n* = 51)	BWT ≤ 3 mm
Civitelli et al. (27), (*n* = 32)	BWT ≤ 3 mm and other IUS features
Castiglione et al. (19), (*n* = 40)	BWT ≤ 3 mm plus absence of hypervascularisation signs
Orlando et al. (29), (*n* = 30)	BWT ≤ 3 mm
Paredes et al. (23), (*n* = 36)	BWT ≤ 3 and 0–1 doppler scale
Castiglione et al. (21), (*n* = 218)	BWT ≤ 3 mm
Zorzi et al. (28), (*n* = 80)	BWT ≤ 3 mm
Ma et al. (26), (*n* = 77)	BWT ≤ 3 mm and normalization of stratification, no hypervascularisation, resolution of mesenteric inflammatory fat, and no complications (IUS)
Calabrese et al. (24), (*n* = 188)	BWT ≤ 3 mm ileum/4 mm for colon, normal Limberg score and no complications
Helwig et al. (25), (*n* = 137)	BWT and Color-Doppler normalization, no loss of stratification and no fibro fatty proliferation

*BWT, bowel wall thickness; IUS, intestinal ultrasound.*

For instance, Civitelli et al. (27) added the complete normalization of vascularization assessed by using Doppler US, normal bowel wall stratification, absence of mesenteric fat hypertrophy, nodes enlargement, and disease complications (i.e., strictures, fistulas). Ma et al. (26) used these same features to define TH.

Calabrese et al. (24) defined TH as the normalization of all the IUS parameters, such as BWT less than or equal to 3 mm, Limberg score normalization, normal wall stratification and absence of mesenteric fat hypertrophy, nodes enlargement, and disease complications (i.e., strictures, fistulas).

More recently, Helwig et al. (25) introduced three definitions of TH: simplified TH, extended TH, and complete TH. The first one accounts for BWT and Doppler features normalization; the second one included BWT normalization and the evaluation of at least two parameters among Doppler US normalization, normal wall stratification, and the absence of fibro-fatty proliferation. Finally, the third definition included the normalization of all four parameters.

### Clinical Implications of Transmural Healing

Several studies showed that the achievement of TH in CD had been associated with significant improvements in clinical outcomes, hospitalization rates, corticosteroid-free remission, treatment intensification, and need for surgery (Table 2).

**TABLE 2:** Relationship between transmural healing and clinical outcomes in patients with Crohn’s disease (CD).

References	Country	Clinical outcomes (*p*-value for correlation with TH)	Time of follow-up	TH response rate	TH assessment method
Castiglione et al. (18), (*n* = 133)	Italy	CR: CDAI < 150 (poor agreement)	2 yo	25%	IUS
Ripollés et al. (22), (*n* = 51)	Spain	CR: HBI < 5 (*p* = 0.001)	12 w–1 yo	29.5%	IUS
Castiglione et al. (19), (*n* = 40)	Italy	CR: CDAI < 150 (poor agreement)	2 yo	25%	IUS/MRE
Orlando et al. (29), (*n* = 30)	Italy	Surgery (*p* = 0.003)	14 w – 1 yo	30%	IUS
Paredes et al. (23), (*n* = 36)	Spain	Steroid use; hospital admission; surgery (global *p* = 0.001)	12 w	42.4%	IUS
Castiglione et al. (21), (*n* = 218)	Italy	Corticoid free remission (*p* < 0.01); longer time to clinical relapse (*p* = 0.01); surgery (*p* < 0.001); hospital admission (*p* < 0.001); need for drug escalation (*p* < 0.001); switch/swap therapy (*p* < 0.01); Clinical outcomes in patients on biologics withdrawal (*p* < 0.001)	12 w	31.2%	IUS
Zorzi et al. (28), (*n* = 80)	Italy	Need of hospitalization (*p* = 0.005); need for steroids *p* = 0.007; surgery (*p* = 0.004)	12–24 mo	32%	SICUS
Ma et al. (26), (*n* = 77)	China	Corticoid free remission (*p* < 0.001); drug escalation (*p* = 0.003); hospital admission (*p* = 0.005); surgery (*p* = 0.03)	12–42 mo	32%	IUS
Calabrese et al. (24), (*n* = 188)	Italy	HBI < 5 (*p* = 0.002)	12 mo	31.2%	IUS
Helwig et al. (25), (*n* = 137)	Germany	HBI < 5 (*p* = 0.001)	12 w	43%	IUS

*TH, transmural healing; CR, clinical remission; CDAI, Crohn’s Disease Activity Index; HBI, Harvey-Bradshaw Index; yo, years; m, months; w, weeks; IUS, intestinal ultrasound; SICUS, small intestine contrast ultrasonography; MRE, magnetic resonance enterography.*

The relationship between clinical remission (CR) and TH has been investigated in five studies; three studies found a significant correlation between TH achievement and CR (22, 24, 25). In a prospective multi-centre longitudinal study conducted by Ripollés et al. (22), 51 patients with CD who underwent therapy with anti-tumour necrosis factor-alpha (TNF-alpha) drugs were followed at 12 and 52 weeks. A total of 29 (56.9%) patients reported an improvement or normalization of sonographic features at 52 weeks and 28 out of 29 (96.5%) patients showed clinical remission according to the Harvey–Bradshaw Index (HBI) (HBI < 5 for remission) or response (HBI decrease >3). Hence, there was a significant relationship between sonographic score and clinical response at 52 weeks. Indeed, sonographic response after 12 weeks predicted clinical response at 1 year, since patients without sonographic improvement were more likely to have a change or intensification in medication or surgery during the year of follow-up.

In a cohort study conducted by our group (19), 80 patients with CD were prospectively enrolled and followed-up for 2 years to assess the rate of TH after starting anti-TNF-alpha therapy.

Overall, 10 out of 40 patients achieved TH (25%) after 2 years. It is noteworthy that there was a good agreement between mucosal healing and TH assessed by IUS (*k* = 0.63, *p* < 0.001) and by MRE (*k* = 0.64, *p* < 0.001). All the patients who achieved TH were in CR even though the agreement between TH and CR was poor (*k* = 0.27; *P* < 0.01).

Subsequently, the same Italian group prospectively analyzed the clinical implications of achieving TH and compared them with those in patients with only mucosal healing or no healing. Overall, 218 patients completed a 2-year treatment with anti-TNF-alpha. A total of 68 (31.2%) patients reached TH measured with BWT ≤ 3 mm at IUS and mucosal healing, while 60 (27.5%) patients had mucosal healing. Importantly, patients who reached TH had a higher rate of steroid-free clinical remission (95.6%), lower rates of hospitalization (8.8%), and need for surgery (0%) at 1 year compared to those who had mucosal healing (75, 28.3, and 10%, respectively) and no healing (41, 66.6, and 35.5%, respectively) (*P* < 0.001). Furthermore, TH was linked to longer intervals until clinical relapse [hazard ratio (HR) 0.87, *P* = 0.01], hospitalization (HR 0.88, *P* = 0.002), and surgery (HR 0.94, *P* = 0.008) than mucosal healing (21).

Similar evidence comes from a study by Zorzi F et al. wherein patients who achieved transmural remission after anti-TNF-alpha treatment did not require surgery, needed fewer steroids, and showed reduced hospitalization rates at 18 months (28).

Furthermore, in a prospective Spanish study, including 36 patients, TH assessed with IUS in the week before starting anti-TNF-alpha treatment, at 12 weeks and 1 year later, was significantly related to better clinical outcomes, i.e., no need to re-introduce corticosteroids or intensify maintenance therapy and/or need for surgery (23).

Notably in a prospective observational study, Orlando et al. (29) assessed the role of ultrasound elasticity imaging in predicting therapeutic outcomes in 30 patients with CD who underwent IUS and ultrasound elasticity imaging at baseline, 12 weeks, and 52 weeks. A BWT < 3 mm was used to define TH. Bowel wall stiffness was evaluated through the strain ratio between the mesenteric tissue and the bowel wall. Severe ileal fibrosis was defined by a strain ratio ≥2. Eight out of 30 patients achieved TH at 14 weeks during the follow-up and one patient achieved TH at 52 weeks. Regarding clinical outcome, the frequency of surgery was significantly higher in patients with a strain ratio ≥2 groups than in <2 groups (*p* = 0.003).

A prospective, longitudinal cohort study (26) conducted in a single tertiary hospital in China investigated the impact of TH assessed with IUS on long-term positive outcomes compared to mucosal healing. Both the mucosal healing and TH were associated with better long-term outcomes based on the univariate analysis, while in the multi-variate analysis, TH was an independent predictor of steroid-free CR [odds ratio (OR), 52.6; *p* < 0.001], drug escalation (OR, 0.1; *p* = 0.002), and hospitalization (OR, 0.05; *p* = 0.005).

More recently, Helwig et al. (25), in a *post hoc* analysis, including 351 patients with IBD belonging to multi-centre studies such as the TRUST and the TRUST&UC, confirmed the positive association between TH and clinical outcomes. Indeed, patients with CD who achieved TH were more likely to reach clinical remission at week 12 [odds ratio (OR), 3.33 (1.09–10.2); *p* = 0.044].

With the aim of measuring therapeutic response, Allocca et al. (30) have proposed an ultrasound score [bowel ultrasound score (BUS)] to best predict the endoscopic activity of CD. It included the following parameters: BWT; bowel wall pattern; bowel wall flow, vascular signals at color Doppler; stricture, fistula, abscess, enlarged mesenteric lymph nodes (short axis >5 mm) and mesenteric hypertrophy. These features have been evaluated for each intestinal segment affected by the disease. The worst segment was selected and used for the BUS calculation. A score of <3.52 was considered predictive of disease remission.

### Clinical Application of Intestinal Ultrasound for Detecting Post-operative Crohn’s Disease Recurrence

The application of IUS in post-surgical CD recurrence has become an essential part of disease monitoring, despite colonoscopy being still considered the gold standard for detecting post-operative recurrence (POR) (31, 32). Early therapeutic intervention is paramount to prevent disease recurrence, as recently suggested (33). However, patients not easily accept and are often reluctant to undergo colonoscopy only a few months after surgery. In this context, IUS could be a surrogate tool to guide early therapeutic strategies and, thereby, the right timing to perform colonoscopy. Several studies have investigated the correlation of IUS and colonoscopy during the first year after CD surgery. A prospective study conducted by our group (34) showed that IUS and small intestine contrast ultrasound (SICUS) had a sensitivity of 77 and 82%, respectively, and they both had a specificity of 94% for POR detection. Subsequently, in a study cohort of 72 patients with CD, Calabrese et al. (35) found a good correlation between BWT (BWT > 3 mm considered predictive of disease recurrence) assessed by SICUS and the Rutgeerts score (RS) (RS ≥ i2 indicating endoscopic recurrence) at colonoscopy (*P* < 0.0001; *r* = 0.67). The same group in 2013 (36) confirmed a good correlation between SICUS and CTE in detecting BWT (*k* = 0.79) and disease extent (*k* = 0.89; *p* < 0.0001), supporting the use of SICUS in routine clinical practice.

In a 5-year experience conducted on 40 post-operative patients with CD, Onali et al. (37, 38) reported the usefulness of IUS for 1-year POR assessment; even though, SICUS was not predictive of clinical recurrence at 4 and 5 years. Subsequently, a meta-analysis (39), including 536 patients, observed a good pooled sensitivity and specificity of IUS for POR detection (sensitivity = 0.94; specificity = 0.84). Of note, a BWT ≥ 5.5 mm was predictive of severe endoscopic POR (Rutgeerts score > i3), so it could be reasonable to manage therapeutic escalation and decisions based on these IUS findings. However, it is noteworthy that colonoscopy cannot be avoided even in patients with a low risk of POR with BWT < 5.5 mm.

More recently, a retrospective study (40), including 201 post-operative patients with CD followed for a median of 7.6 years, showed that IUS recurrence (defined as either anastomotic BWT > 4 mm or new abdominal complication) predicted surgical recurrence, i.e., new major abdominal surgery or symptoms not controlled by medical treatments, while endoscopic recurrence was not predictive of late clinical recurrence (after 36 months) and, thus lacks prognostic value.

Carmona et al. (41) conducted a retrospective observational study on 31 patients with CD who previously underwent ileocecal resection. They found a sensitivity and specificity of 100% and 86.6% for BWT of 3.4 mm with the area under the receiver operating characteristic (AUROC) curve of 92.9%. Based on these findings, IUS represents a feasible and reliable alternative to colonoscopy, especially in early stage of surgery.

Further non-invasive methods such as faecal calprotectin (FC) have been shown to be accurate for the assessment of POR. In a prospective cohort study, Lopes et al. (42) reported a good correlation between increased FC and POR based on modified Rutgeerts score (MRS – MRS > i2b defining POR). Of note, the AUROC for FC cut-off value of 100 μg/g was 0.831 (*p* < 0.05).

In a systematic review by Tham et al. (43), FC 150 μg/g was considered the best cut-off value for predicting POR (defined as a RS > i2) with a pooled sensitivity and specificity of 70% and 69%.

However, large prospective studies are needed to determine how IUS can be collocated in the algorithm of POR and explore the magnitude of benefit of combined IUS and FC for detecting POR and establishing an optimal FC threshold.

### Clinical Application of Intestinal Ultrasound Combined to Non-invasive Markers

In recent years, we have seen the advent of a new era of non-invasive monitoring of IBD.

The use of biomarkers, such as C-reactive protein (CRP) and FC, is more and more expanding in clinical practice. CRP is the most widely used inflammatory marker and it has been shown a strong correlation with CD activity, while less correlation with UC.

A possible explanation could be that in UC, the inflammation is limited to the mucosa unless very severe, whereas, in CD, it is extended to all the bowel wall layers (44). However, CRP normalization does not predict a complete clinical and endoscopic remission (45–47).

The correlation between CRP levels and TH was investigated in 2013 by our group (18) in a longitudinal observational study, including patients with CD. There was a good concordance between TH and CRP levels (*k* = 0.79; *p* = 0.02). Subsequently, these results were confirmed in 2017 (19) (*k* = 0.77; *p* = 0.02).

Despite practical concerns about variability, lack of standardized cut-off for disease activity and patients’ reluctance to collect stool samples serially, FC is now routinely used in clinical practice for monitoring disease activity and response to medication (45, 48).

The trial “Effect of Tight Control Management on Crohn’s Disease (CALM)” has paved the way for tight monitoring, including FC measurement (49). When compared with patients in the standard of care group, the treat-to-target group achieved better outcomes, such as mucosal healing, deep remission, and less need for steroids.

Weinstein-Nakar first explored the diagnostic accuracy of FC for predicting deep remission assessed by MRE in a pediatric population. A cohort of 243 patients showed that FC had the AUROC 0.93–0.94 for predicting deep healing, defined as mucosal healing [Simple Endoscopic Score-CD (SES-CD) 0–2] plus TH on MRE. Deep healing resulted in lower FC levels [median 10 μg/g, interquartile range (IQR) 10–190] than endoscopic healing or TH alone, with optimal cut-offs of 300 μg/g and 100 μg/g for mucosal healing and deep healing, respectively (50).

In a retrospective study, 268 Korean patients diagnosed with CD and treated with anti-TNF-alpha agents underwent colonoscopy, radiological assessment and FC measurement in 3 months. They found that an FC cut-point level of 81.1 mg/kg predicted deep healing, defined as the combination of endoscopic and radiological healing, with a sensitivity of 0.623 and a specificity of 0.817 (AUROC, 0.767; 95% CI, 0.702–0.832). The fecal calprotectin AUROC increased to 0.805 when serum albumin and CRP were added to the evaluation (95% CI, 0.752–0.858) (51).

However, there are limited data about IUS and FC. Calabrese et al. (24) explored the correlation between TH and combined CRP and FC. They found that a significant (*p* < 0.002) proportion of patients achieving TH had clinical remission and normalization of CRP and FC assessed by using qualitative measurement at 3, 6, and 12 months compared to the baseline.

In the *post hoc* analysis of the TRUST study (25), patients with CD who achieved TH at week 12 reported improved CRP and FC levels, even though this was not significant compared with patients without TH, defined as no IUS improvements.

With regards to UC, Maaser et al. (52), in the prospective, observational TRUST&UC study conducted at 42 German IBD-specialized centres, found that an FC normalization (intended as a value >250 μg/g becoming <250 μg/g at week 12) positively correlates to a BWT normalization in sigmoid (*p* = 0.023) and descending colon (*p* = 0.029).

Furthermore, in a single Italian study, Allocca et al. (53) investigated the diagnostic accuracy of IUS and/or FC compared with colonoscopy (CS) in assessing endoscopic activity. The sensitivity and specificity of BWT > 3 mm or FC > 101 μg/g was 100 and 53%, respectively, for endoscopic disease activity, while when taken into account together, they resulted in a sensitivity of 84 and specificity of 93%.

Regardless, more studies are awaited to integrate IUS, CRP, and FC into the definition of deep healing.

### Intestinal Ultrasound and Ulcerative Colitis: Ready for Use?

The application of IUS in UC is less well established since endoscopic evaluation and histology have been considered the critical stakeholders for assessing disease extent and severity.

However, in the setting of tight monitoring, IUS has become the focus of current intense study (54), even in UC, due to the need for non-invasive, easily available tools.

Growing evidence suggests that its use is reliable, objective and well accepted because it could reduce the need for colonoscopy with related risks, potential complications, sedation, and bowel preparation.

Currently, the role of IUS in UC could be to evaluate disease activity and disease extension.

Assessment of BWT is the most reliable IUS measure. A recent expert panel identified several key IUS features complementary to the bowel wall thickness, such as parietal blood flow, Doppler signal, wall layer stratification, and fatty wrapping. They further assessed the reliability of IUS parameters among expert sonographers, and it is noteworthy that inter-observer agreement was almost perfect for BWT [intra-class correlation coefficient (ICC): 0.96] and substantial for color Doppler signs (κ = 0.63) (55).

Several studies found also differences between different colonic segments and the other age groups of patients (56). Maaser et al. (52), in the TRUST&UC study, identified a threshold of 4 mm for the sigmoid colon and 3 mm for descending, transverse, and ascending colons for defining active disease. In a prospective multi-centric study, Kinoshita et al. (54) described four grades of severity according to the presence of the following features: the first one consisted of normal wall colonic thickness; the second one consisted of thickened mucosa and sub-mucosa without hypoechoic change of the sub-mucosa; the third one consisted of bowel wall thickness with loss of stratification; the last one consisted of bowel wall thickness with loss of stratification and irregular mucosa.

Regarding UC extension, the overall accuracy of IUS in terms of BWT has been acceptable compared to endoscopy. In addition, when BWT was combined to color Doppler assessment, the accuracy increased, and it has been reported around 95% across all the bowel segments measured.

A recent systematic review highlights a significant heterogeneity between all the IUS measures for assessing disease activity and identified that increased BWT and detection of increased blood flow by color Doppler were the most often applied criteria for disease activity and distribution (56).

In a prospective observational study, including 53 patients with UC, Allocca et al. (53) reported that BWT > 3 mm, hypoechogenicity, a signal on power Doppler, and lymphadenopathy correlated with endoscopic disease activity. Based on them, a score consisting of BWT > 3 mm plus vascularity or flow within the colonic wall or BWT > 4.43 mm alone without vascular signal was built. This score had a sensitivity of 0.71 and a specificity of 1.00 in assessing disease activity and inter-observer agreement was excellent (κ = 0.86) so it has been recently validated under the name ‘Milan ultrasound criteria (MUC)’ (57) in a cohort of 98 patients with UC. Importantly, there was a strong correlation between MUC and the Mayo Endoscopic Score (MES) at baseline (*p* = 0.653; *p* < 0.001). In addition, a baseline MUC > 6.2 was predictive of a negative disease course (HR: 3.87, 95% CI: 2.25–6.64, *p* < 0.001), while patients with MUC < 6.2 had a significantly lower cumulative probability of treatment escalation, need of corticosteroids, hospitalization, and colectomy (58).

Similarly, Bots et al. (59) developed and internally validated, in 60 patients with UC, a new index for grading disease activity, using endoscopy with MES as the reference standard. Based on this index, a BWT > 2.1 mm discriminated between remission (MES 0) and mild (MES 1) endoscopic activity, while a cut-off of 3.2 mm distinguished between mild and moderate endoscopic activity (MES 0–1 vs. MES 2–3) and, finally, a BWT > 3.9 mm correlated with severe endoscopic activity. The other parameters included in this score were an enhanced color Doppler signal and a lack of haustrations, both predicting disease activity. In addition, fat wrapping was predictive of severe disease.

The TRUST&UC study (52) has shown that BWT correlates with disease clinical activity scores measured using the Simple Clinical Colitis Activity Index (SCCAI) at the beginning of a flare and after 12 weeks of therapy. High BWT (> 4 mm in the sigmoid colon and >3 mm in the other segments) and the Doppler signal were observed at the time of diagnosis and over 12 weeks (at 2, 6, and 12 weeks). Loss of haustration, loss of wall stratification, ascites, lymphadenopathy, and mesenteric fat proliferation were further evaluated during follow-up. It is noteworthy that the percentage of patients with high BWT changed significantly over time from baseline to 12 weeks after starting therapy and BWT at week 2 predicted the response to medical treatment followed by the SCCAI.

Kinoshita et al. (54) performed the first prospective multi-centric study to compare US with colonoscopy for assessing UC. They found a significant overall correlation between US and colonoscopy in all the colonic segments (*k* = 0.55; *p* < 0.001) and the concordance for each colonic segment was moderate, whereas, for the rectum, it was poor.

The poor diagnostic accuracy of IUS for detecting inflammation in the rectum in patients with UC was further confirmed by a recent meta-analysis (60). Good sensitivity and specificity were reported for detecting active disease (when BWT > 3 mm) in the right and transverse colons. However, this accuracy decreased toward the rectum due to the rectum’s deep position in the pelvis and to the small intestine gas distension.

Hence, IUS is not considered a reliable tool for assessing rectal disease. Sagami et al. (48) used the IUS combined with transperineal ultrasound (TPUS) and FC for active patients with UC who required colonoscopy as the gold standard to evaluate the rectum. BWT < 4 mm in TPUS was a significant independent predictor for rectal endoscopic and histologic healing (*p* < 0.05) and, notably, the predictability was better than FC. Thus, the authors proposed TPUS in combination with IUS to assess the whole colon (61).

## Future Directions

Current evidence suggests that IUS is shifting from a tool to diagnose IBD and simply discriminate active vs. inactive disease to a more complex, valid, and reliable instrument for closely monitoring patients with IBD and predicting therapeutic response.

Recent evidence supports that adjusting therapeutic approaches based on IUS parameters appear to be a reasonable strategy in CD. Indeed, assessing IUS findings after starting therapy could be crucial to establish the early response to treatment and speed up making clinical decisions.

The use of IUS in UC is still challenging, given that endoscopy and, more recently, histology remain the reference standard for assessing UC activity. However, based on the latest findings, it is time to include IUS in routine clinical management.

Developing IUS scores to determine disease activity and response to treatment is the next crucial step toward standardized IBD monitoring and expanding IUS adoption.

We strongly believe that in the next future, the full acknowledgment of the role of IUS in IBD could allow us to replace invasive assessment of endoscopic response/remission and thus reduce the psychological burden of colonoscopies on the patients.

## Author Contributions

OMN and GC contributed to the conception, design, drafting, and revision of the manuscript. AT, AC, and GF contributed to the manuscript draft. AR critically reviewed the manuscript. FC contributed to the conception and critically reviewed the manuscript for important intellectual content and provided overall supervision. All authors have contributed to the article and approved the submitted version of the manuscript.

## Conflict of Interest

The authors declare that the research was conducted in the absence of any commercial or financial relationships that could be construed as a potential conflict of interest.

## Publisher’s Note

All claims expressed in this article are solely those of the authors and do not necessarily represent those of their affiliated organizations, or those of the publisher, the editors and the reviewers. Any product that may be evaluated in this article, or claim that may be made by its manufacturer, is not guaranteed or endorsed by the publisher.
